# Accelerating Actions Against Malnutrition: A Call for Strengthening the Capacity of Health and Nutrition Program Staff in Devbhumi Dwarka, Gujarat

**DOI:** 10.7759/cureus.28616

**Published:** 2022-08-31

**Authors:** Mrunal Mehta, Somen Saha, Apurvakumar Pandya, Mayur B Wanjari, Deepak Saxena

**Affiliations:** 1 Public Health, Indian Institute of Public Health, Gandhinagar, IND; 2 Public Health, Jawaharlal Nehru Medical College, Datta Meghe Institute of Medical Sciences, Wardha, IND; 3 Parul Institute of Public Health, Parul University, Vadodara, IND; 4 Epidemiology and Public Health, Jawaharlal Nehru Medical College, Datta Meghe Institute of Medical Sciences, Wardha, IND

**Keywords:** integrated child development services, india, gujarat, health & icds program, malnutrition, training needs assessment

## Abstract

Background

The Integrated Child Development Services (ICDS), a flagship program of the Government of India, is addressing the malnutrition, health, and development needs of young children, pregnant and lactating women, and adolescent girls for more than four decades. Although the program has been implemented for the past four decades, it could not bring the expected outcomes in terms of reducing malnutrition. The program's limited success can be attributed, among others, to insufficient skills of the program staff and inadequate convergence with the existing nutrition programs implemented through the health department. For the success of any program, advanced knowledge, improved skills, motivation, and the right attitude of the program staff are essential and can be instilled through the need-based training of the staff. The present study aimed at identifying gaps in existing training for health and ICDS program staff in the district and developing a capacity-building strategy to strengthen the implementation of the nutrition program in the district, including "Project Tushti," which aimed at combating malnutrition in Devbhumi Dwarka district of the Gujarat state.

Methods

The training needs assessment (TNA) was conducted using a descriptive cross-sectional study design. TNA is a method to determine program gaps and training required to fill in programmatic gaps. Appropriate use of TNA can promote designing effective training and nurture program staff productivity, thereby ensuring efficient use of resources for achieving desired program outcomes within the prescribed timeline. Considering the coronavirus disease 2019 (COVID-19)-led lockdown, convenient sampling was used to reach out to potential study participants. A total of 150 program staff from both health departments (particularly medical officers, taluka health officer, National Adolescent Health Program - Rashtriya Bal Swasthya Karyakram (RBSK) medical officer, community health officer from health and wellness center, female health workers, and Accredited Social Health Activists (ASHAs)) and ICDS team (chief district program officer, supervisor, Anganwadi workers, and helpers at Anganwadi center) were interviewed telephonically using a semi-structured interview guide. Interviews were conducted between May 10 and 16, 2020.

Results

Results reveal that about 49% of the health team and ICDS staff had not received nutrition-specific training in the last year. In terms of coverage, the training coverage was partial, and training content on nutrition was limited. Training contents were divided based on supervisory and implementing cadre feedback. Participants expressed the need for in-depth nutritional refresher training, including topics such as community-based management of acute malnutrition, identification of malnutrition, use of ICDS common application software, and soft skills such as communication skills and supportive supervision. Although 62% of participants preferred the face-to-face medium of training, they agreed with the online mode of training.

Conclusion

Findings indicate an urgent need for training, particularly in the district's nutrition domain for the staff involved in the implementation, and soft skills for supervisory level staff. Innovative training approaches using the digital platform can be explored for training delivery. The paper discusses innovative capacity-building strategies to address training needs effectively.

## Introduction

The Integrated Child Development Services (ICDS) is one of the oldest, biggest, and flagship community-based programs of the Government of India. The program is aimed at addressing malnutrition, health, and development needs of young children, pregnant and lactating women, and adolescent girls. It has been in place for more than four decades, 47 years to be precise; however, its impact on reducing malnutrition is limited. The program's limited success can be attributed, among other reasons, to insufficient sustainable capacities within implementing organizations and communities. For the success of any program, advanced knowledge, improved skills, motivation, and the right attitude of the program staff are essential ingredients.

Behavioral factors of both beneficiaries and the community significantly affect the prevalence of malnutrition [1,2]. It is noted in the literature that lack of knowledge and adequate skills prevent providing timely and correct inputs to the interventions, which delays achieving expected nutrition program outcomes [3,4]. Training remains an important medium to impart knowledge and skills and instill motivation and the right attitude [5,6]. The capacity building of the program staff contributes to achieving desired program outcomes [7,8].

The Government of India has now emphasized strengthening the ICDS program's training component to improve the service delivery mechanism and accelerate better program outcomes [8]. Training modules are standardized, missing out on the local context. Even translated modules lack local context. Most recent modules are focused on ICDS programs while updated modules on nutrition for health staff are not yet available. In all existing modules, topics such as soft skills, behavior change communication, negotiation skills, documentation skills, use of digital tools, and smartphones are not covered.

The Ministry of Women and Child Development, Government of India [8], has developed training resource centers (middle-level training centers and Anganwadi training centers) across the country and developed training guidelines and modules on ICDS. This is a traditional approach where participants need to gather at the designated training center for the training. This can be one major obstacle in the current coronavirus disease 2019 (COVID-19) pandemic and post-pandemic context due to the need for social distancing and protection of people from coronavirus infection. The National Institute of Public Cooperation and Child Development (NIPCCD) [9] has developed e-courses for ICDS functionaries (child development program officer (CDPO), supervisors, and Anganwadi workers (AWW)), refresher courses for CDPO, as well as thematic e-courses such as infant and young child feeding, integrated child development, and basic nutrition. Recently, the government has initiated the Integrated Government Online Training (I-GOT) Program [10]. The Public Health Foundation of India (PHFI) [11] has also introduced a six-month-long e-course for the ICDS supervisory cadre with an aim to update current knowledge, strengthen the technical capacity on reproductive and child health nutrition, and enhance the managerial skills of the service providers and officials working under the ICDS program.

Despite extensive training programs, the challenge remains in delivering health and nutrition services to children, pregnant and lactating women, and adolescent girls. For example, in the current ICDS program, children from birth to three years and lactating women are missing, as most services are provided at Anganwadi center where children from three to six years, adolescent girls, and pregnant women are catered. Anganwadi workers are not as efficient as required in delivering home-based child care for children below three years. These training and e-courses are in English and do not address local field challenges. Inadequate skills of community-based workers, poor supervision, weak monitoring and evaluation of logistical gaps, resource scarcity, and poor utilization continue to hamper progress and affect the program's outcome [12-16]. Therefore, for any program to be effective, designing a capacity-building strategy based on training needs assessment (TNA) is crucial for achieving the program's expected outcomes. The present study aimed at understanding the current training status of the health and nutrition program staff in the district, identifying training gaps in the domain of nutrition, and understanding the preferred medium of training to formulate innovative and targeted training interventions. The goal of the TNA was to identify gaps in existing training for health and ICDS program staff in the district and to develop a capacity-building strategy to strengthen the implementation of the "Tushti project," which aimed to combat malnutrition in the Devbhumi Dwarka district of the Gujarat state in India.

## Materials and methods

The TNA was conducted using a descriptive cross-sectional study design. TNA is a method to determine program gaps and training required to fill in programmatic gaps. Appropriate use of TNA can promote designing effective training and nurture program staff productivity, thereby ensuring efficient use of resources for achieving desired program outcomes within the prescribed timeline.

The role of the health department team is to screen targeted beneficiaries and provide treatment to malnourished persons whereas nutrition education, supplements, and nutrition counseling are provided at Anganwadi centers. Both programs are operated parallelly; hence, the program staff of the health department and ICDS need to work closely with each other. However, there have been gaps in the achievements of the nutrition outcomes of the project staff and ICDS team. TNA was carried out to know the training requirements of different cadres with the health department and ICDS and develop a strategic training plan for the district. Considering the COVID-19-led lockdown, convenient sampling was used to reach out to potential study participants. Study participants were telephonically interviewed using a survey questionnaire. Interviews were conducted between May 10 and 16, 2020.

Out of a total of 2,441 personnel employed with ICDS and the health department in Devbhumi Dwarka, 263 team members (226 from the health department and 37 from ICDS) were identified for inclusion in the study. Participants included child development program officers (CDPOs) and Mukhya Sevika (MS) from the ICDS program whereas Rashtriya Bal Swasthya Karyakram (RBSK) medical officer (MO), female health worker (FHW), and community health officer (CHO) were from the health department contributing to the nutrition program in the district. All of them were contacted telephonically and 150 staff (57%) participated in the study. Table 1 depicts a selection of study participants.

**Table 1:** Inclusion and exclusion criteria for the study

Inclusion criteria	Exclusion criteria
Staff who are from the health department providing nutrition services (primarily Rashtriya Bal Swasthya Karyakram and Rashtriya Kishor Swasthya Karyakram) and the Integrated Child Development Services (ICDS) team	Staff members who were involved in COVID-19 duties
Working for at least two years in the district	Not able to contact after third call attempt/not able to talk due to network issues, or phone disconnected in between and not able to reconnect
Working for at least one year in a nutrition program	Transfer to another district
Able to speak in Gujarati	Given additional charge of the nutrition program in last six months
Willing to participate in the telephonic interview	Does not know Gujarati

The semi-structured survey questionnaire was developed and validated by a panel of experts. Experts included faculty from the Indian Institute of Public Health Gandhinagar (IIPHG), a senior district health official, an ICDS officer, a public health intern, and project staff working with ICDS at IIPHG. Expert feedback was incorporated and the tool was finalized. The tool included questions related to the work experience, education, designation, training received so far, training needs (thematic domain and skills), and preferred medium of the training. The tool is attached in the Appendix. Experts from the field who provided their inputs to the tool were excluded from the study.

Ethical consideration

The study was approved by the Institutional Ethics Committee of the Indian Institute of Public Health Gandhinagar (IEC/IRB approval number: 14/2019-20).

## Results

As illustrated in Table 2, about half of the participants (50%) had graduated from the university, 5.3% had post-graduation, and nearly 42.7% had completed the Auxiliary Nursing Midwifery (ANM) course. Nearly 42.7% of participants had more than five years of experience, whereas about 28% had one to five years of experience.

**Table 2:** Profile of study participants * Percentage calculated from row total. RBSK MO: Rashtriya Bal Swasthya Karyakram medical officer; CHO: community health officer; FHW: female health worker; CDPO: child development program officer; MS: Mukhya Sevika.

Cadre	Total participated in the study	Education, N (%)						Experience, N (%)			
		12th	Auxiliary Nursing Midwifery course	Graduation (Ayurved/homeopath/nursing/humanities)	Post-graduation (nursing/humanities)	Total	<1 year		1-5 years	>5 years	Total
RBSK MO	17 (11.3)	0	0	17 (22.7)	0	17	9 (52.9)		5 (11.9)	3 (4.7)	17
CHO	8 (5.3)	0	0	8 (10.7)	0	8	8 (100)				8
FHW	91 (60.6)	0	64 (42.7)*	24 (32)	3 (37.5)	91	18 (19.8)		27 (64.3)	46 (71.9)	91
CDPO	6 (4)	0	0	5 (6.7)	1 (12.5)	6	2 (33.3)		1 (2.3)	3 (4.7)	6
MS	28 (18.6)	3 (10.7)*	0	21 (21)	4 (50)	28	7 (25)		9 (21.5)	12 (18.8)	28
Total	150	3 (2)	64 (42.7)	75 (50)	8 (5.3)	150	44 (29.3)		42 (28)	64 (42.7)	150

Major training domains included ICDS overview, Early Childhood Care and Education (ECCE) training, incremental learning, Mukhya Sevika work training and refresher training, Infant and Young Child Feeding (IYCF) practices, CDPO training, and Anganwadi workers training.

Nearly half (49%) of the staff had not received nutrition-specific training in the last one year. Of those who received any training (51%), coverage of the training was partial (training of all essential domains was not covered); MS (41%) followed by FHW (33%) had received the training, whereas participation of RBSK MO (7%) and CHO (6%) was limited.

Out of four blocks in Devbhumi Dwarka, the staff representation in training was primarily from Dwarka and Bhanwad blocks. Across all training, more than half of the MS (54%) were from the Dwarka block, whereas 46% FHWs were from the Bhanwad block. Participation from the other two blocks was negligible. Most RBSK MOs (68%) who participated in the training were from Khambhaliya and Dwarka blocks, whereas most CHOs (89%) were from only one block, Khambhaliya. This indicates that the participation of health and nutrition staff in the training was not harmonious.

The training content was limited to the basics of the nutrition program structure and its delivery. Of the participants, 68% expressed the need for in-depth refresher training on nutrition. Training on community-based management of acute malnutrition, identification of malnutrition, and use of software were important topics for future training. In recent training modules, training on soft skills such as behavior change communication skills, team-building, and the use of reporting software was missing. Of the participants, 79% expressed the need for training on supportive supervision, behavior change communication, team building, and conflict management.

Of the participants, 62% preferred the online mode for the training, especially in the context of the COVID-19 pandemic, followed by periodic face-to-face training. Nearly a quarter (21%) desire to receive training exclusively online.

## Discussion

There is an urgent need for training, particularly in the nutrition domain, and supportive supervision of supervisor-level employees in the district. The training assessment conducted in Odisha highlighted the gaps in terms of transfer of knowledge from supervisors to AWWs and AWWs applying the learning in their work [14].

The present study highlighted limited coverage of the training. An evaluation study conducted in Gujarat indicated poor training may be a reason for poor program outcomes [15]. All the trainees felt inadequate of the lack of a follow-up plan to keep the learning intact, training timing, facilitation skills of the trainers, availability of appropriate audio-visual aids, and training material [14-16].

Additionally, in the present study, training on soft skills such as behavior change communication skills, team-building, and the use of reporting software was missing. Participants expressed the need for training on the above-mentioned topics. Developing the capacity of the program staff is an essential step for strengthening overall program outcomes [17-18].

Despite the well-conceptualized ICDS program and training mechanism, need-based training delivery and capacity building continue to hamper the program's progress. There is a need to facilitate appropriate training and handholding of the supervisors and their managers, both from the health and ICDS programs equally, to empower them with knowledge, appropriate skills, and motivation to contribute to improving the program's implementation by training field staff and implementing strategies in their supervisory work.

Innovative training approaches using a digital platform that addresses local needs can be explored as 62% of participants agreed to the provision of online training, especially in the present context of the COVID-19 pandemic. A study [18] conducted in the Wardha district in 2017 found that an online training program for frontline health workers (FHW) was quite feasible and well accepted by FHWs. Thus, the feasibility and acceptability of online training are optimal. Mobile applications have recently emerged as a potential tool for delivering online training. Mobile applications help to schedule, provide training, and extend follow-up/supportive supervision support to field staff.

Proposed strategic actions for capacity building

Based on the training needs assessment, there is a need for more programmatic and solution-based training for various levels of ICDS functionaries to improve the service delivery mechanism and accelerate better program outcomes. These approaches are discussed below.

Scaling Up the Umbare Anganwadi Initiative

Umbare Anganwadi (means Anganwadi at doorstep), an initiative under the ICDS program of Gujarat during the COVID-19 pandemic, was designed to aid continuity of behavior change communication at a time when Anganwadi centers were closed. The ICDS program also initiated a television program with regular theme-based modules in an interactive way. Such initiatives should be scaled, including using mobile-based applications to promote good health and nutrition that can act as a "Poshan Setu" or bridge to close the gaps in malnutrition.

Mobile-Based Training Application

Training-based mobile applications can be developed based on imparted training modules, which will help the functionaries access every time, which will be offline. The training application will capture the training sessions conducted, video sharing, question & answer part, and interactive sessions, which will be a learning and sharing platform. Training materials like job aids can be provided in soft copies and periodic training follow-up meetings will help monitor the usage of the training in the field and its impact on program outcomes.

Training Materials in the Local Context

Relevant materials for supervisors and frontline workers are needed for their understanding, mentoring, and sharing with community members. A series of short cartoon booklets along with broad topics, which are easy to understand, locally relevant, and context-driven, should be developed with the active participation and input of supervisory staff.

Limitation of the study

The assessment was conducted with 150 health and ICDS program staff in the Devbhumi Dwarka. The findings of the study were limited to needs as expressed by staff from one district of Gujarat. The generalizability of the findings beyond the district has to be done with caution and will need further studies. In addition, training needs are an evolving issue and have to be done specifically to the context.

## Conclusions

In conclusion, the need for training of health and ICDS program staff working on malnutrition in the district can be addressed by an innovative training approach through the digital platform. This training approach should be aligned with the Poshan Abhiyan run by the Government of India to avoid duplication. Existing training materials developed under the Poshan Abhiyan should be adapted and necessary training materials should be developed as per the need and local context. Concurrent monitoring of capacity-building efforts should be prioritized. The potential of the supervisory cadre from health and nutrition to positively influence the program delivery and consequently improve the nutritional status of children, adolescents, and pregnant and lactating mothers is enormous in accelerating efforts to address malnutrition.

## Appendices

Training needs assessment tool

1. Have you received nutrition-specific training in the last one year?

2. If yes, what was the content of the training, the duration of the training, and how frequently such training was organized?

3. What are the thematic areas within nutrition that you think you need training in?

4. What should be the content and duration of the training?

5. What should be the preferred mode of training - face-to-face or online? Why?

6. What are the areas of your job in which you would like to receive further training or instruction to effectively implement the program?

7. Any other suggestions?
